# Emergence of Hybrid Resistance and Virulence Plasmids Harboring New Delhi Metallo-β-Lactamase in *Klebsiella pneumoniae* in Russia

**DOI:** 10.3390/antibiotics10060691

**Published:** 2021-06-09

**Authors:** Polina Starkova, Irina Lazareva, Alisa Avdeeva, Ofeliia Sulian, Darya Likholetova, Vladimir Ageevets, Marina Lebedeva, Vladimir Gostev, Julia Sopova, Sergey Sidorenko

**Affiliations:** 1 Pediatric Research and Clinical Center for Infectious Diseases, 197022 Saint Petersburg, Russia; sps_96@mail.ru (P.S.); partina-irina@yandex.ru (I.L.); dary.spb@gmail.com (D.L.); ageevets@list.ru (V.A.); guestvv11@gmail.com (V.G.); 2 National Research Institute of Information Technologies, Mechanics and Optics, 191002 Saint Petersburg, Russia; 3 Department of Microbiology, Saint Petersburg State University, 199034 Saint Petersburg, Russia; avdeenko-alya@mail.ru; 4 Saint Petersburg State Academy of Veterinary Medicine, 196084 Saint Petersburg, Russia; sulyan1994@mail.ru; 5 Bryansk Interregional Veterinary Laboratory, Suponevo, 241520 Bryansk, Russia; lemasha117@mail.ru; 6 Center of Transgenesis and Genome Editing, Saint Petersburg State University, 199034 Saint Petersburg, Russia; sopova@hotmail.com; 7 Laboratory of Plant Genetics and Biotechnology, Saint Petersburg Branch of Vavilov Institute of General Genetics, 119991 Saint Petersburg, Russia; 8 Department of Medical Microbiology, North-Western State Medical University named after I.I. Mechnikov, 195067 Saint Petersburg, Russia

**Keywords:** *Klebsiella pneumoniae*, *bla*_NDM_, hybrid resistance, virulence plasmids

## Abstract

The emergence of carbapenem-resistant hypervirulent *Klebsiella pneumoniae* (CR-hvKp) is a new threat to healthcare. In this study, we analyzed nine CR-hvKp isolates of different sequence-types (ST) recovered from patients with nosocomial infections in two hospitals in Saint Petersburg. Whole-genome sequencing showed that eight of them harbored large mosaic plasmids carrying resistance to carbapenems and hypervirulence simultaneously, and four different types of hybrid plasmids were identified. BLAST analysis showed a high identity with two hybrid plasmids originating in the UK and Czech Republic. We demonstrated that hybrid plasmids emerged due to the acquisition of resistance genes by virulent plasmids. Moreover, one of the hybrid plasmids carried a novel New Delhi metallo-beta-lactamase (NDM) variant, differing from NDM-1 by one amino acid substitution (D130N), which did not provide significant evolutionary advantages compared to NDM-1. The discovery of structurally similar plasmids in geographically distant regions suggests that the actual distribution of hybrid plasmids carrying virulence and resistance genes is much wider than expected.

## 1. Introduction

*Klebsiella pneumoniae* (Kpn) is one of the leading causative agents of hospital- and community-acquired infections worldwide. Multidrug-resistant (MDR) and hypervirulent (hvKP) genetic lineages, as well as a recently emerging trend toward their convergence, are the greatest dangers associated with Kpn [1]. Among MDR genetic lineages, the greatest threat is associated with resistance to carbapenems due to the production of carbapenemases. Metallo-β-lactamases (MBLs) are of particular concern since they are insensitive to clinically available inhibitors, including diazobicyclooctanes, thereby limiting the therapeutic options [2].

There are several evolutionary pathways for the formation of Kpn genetic lineages that demonstrate high virulence and resistance to carbapenems, all of which are associated with horizontal gene transfer. Two of these pathways are related to the acquisition of additional plasmids through MDR or hvKP isolates. According to [3], MDR Kpn is more likely to acquire virulence genes than hvKP is to acquire drug-resistance genes due to the ability of MDRs to easily acquire additional genes. However, the majority of carbapenem-resistant hvKP (CR-hvKP) reported to date emerged because of the acquisition of carbapenemase-encoding plasmids (mainly *bla*_KPC-2_ and *bla*_NDM-1_) by hvKP [4]. The reason for the prevalence of this evolutionary pathway is probably the fact that virulence plasmids like pLVPK are nonconjugative which is confirmed by their predominant distribution within a limited number of genetic lines.

Since New Delhi metallo-beta-lactamase (NDM) was first reported in Sweden [5], 31 variants of NDM-type carbapenemases have been identified. The evolution of NDMs results from amino acid substitutions (AAS) or insertions that possess different hydrolytic activity toward antibiotics, which affects the treatment strategy [6]; hybrid plasmid formation and evolution of resistance probably occur simultaneously. Thus, nowadays NDM variants are evolving towards enhanced stability, Zn(II) binding affinity [7], or the ability to function as mono-zinc enzymes with high catalytic efficiency [8].

In a previous study, we described the emergence of Kpn sequence type (ST) 395 and ST147 isolates in an oncology hospital carrying virulence and *bla*_NDM-1_ genes simultaneously; however, we were unable to pinpoint the location of these genes [9]. In another study from Russia, the authors were also unable to accurately determine the location of the resistance and virulence genes [10]. Shaidulina et al. used Illumina MiSeq and ONT MinION systems and succeeded in localizing the virulence genes and *bla*_OXA-48_ on different plasmids. To the best of our knowledge, no reports of CR-hvKp isolates with confirmed hybrid plasmids coharboring genes of carbapenemases and virulence genes have been published in Russia so far [11]. In the present study, we aimed to describe the emergence of Kpn isolates of different STs (ST15, ST147, ST395, and ST874) with hybrid plasmids co-harboring virulence genes and *bla*_NDM_ in the hospitals in St. Petersburg. Moreover, in one of the isolates, *bla*_NDM_ was identified as a new variant (*bla*_NDM-29_).

## 2. Results

### 2.1. Clinical Manifestations and Antimicrobial Susceptibility of Recovered Isolates

Eight hypermucoviscous *bla*_NDM_-positive Kpn isolates, and one hypermucoviscous but *bla*_NDM_-lacking isolate were included in the study (Table 1). One isolate was recovered from a patient in a general hospital and six from patients in an oncology hospital. All oncology patients had solid tumors. Three patients experienced liver failure. The clinical details of the patients are shown in Supplementary Material Table S1. Most isolates were obtained from sites that were not associated with invasive diseases. Two isolates attributed to different clonal lines were recovered from one patient.

Based on multilocus sequence typing (MLST), three Kpn isolates belonged to the ST147-KL20 (CC147) lineage, four to ST395-KL2 (clonal group (CG)395), one to ST874-KL45 (CG258), and one to ST15-KL19 (CG15). Several characteristics of 1657_kpn, 1659_kpn, 1971_kpn, and 2024_kpn isolates, including resistome, virulome, attribution to ST, replicon, K types, LD_50_, and others, are partly represented in our previous study [9]. Detailed genotypic characteristics of isolates are presented in Supplementary Material Table S2. 

The Kpn isolates were resistant to all the tested cephalosporins, carbapenems, fosfomycin, ceftazidime/avibactam, and a trimethoprim–sulfamethoxazole combination, but were susceptible to polymyxin, tigecycline, and aztreonam/avibactam. In addition, they showed miscellaneous resistance to aminoglycosides. The antimicrobial agents and phenotypes of all the tested isolates are listed in Supplementary Material Table S3.

### 2.2. General Sequencing Results

On average, 69,569 reads were generated by Oxford Nanopore with a base call accuracy of Q11 per isolate (average read length: 3.5 kbp). The output from the Illumina platform was, on average, 1,354,011 paired-end reads (average read length: 300 bp) with a sequencing quality of Q36. The hybrid assemblies provided an average of 145.96-fold coverage. The contig number (>1000 bp) of the latter ranged from three to six and yielded an average total length of 5,875,483 bp, with the longest corresponding to a chromosome (approx. 5.3 Mbp). The average N50 size for all isolates was 5,019,528 bp, and the mean GC content was 56.6%.

### 2.3. Plasmids

All the studied isolates carried one to four plasmids, including a large (~311–422 kb) virulence plasmid. Sequence analysis revealed that these multireplicon plasmids were a fusion of IncFIB and IncHI1B backbones and carried both antimicrobial resistance (AMR) and virulence genes. The phvKpST395_2024 and phvKpST395_NDM-1_1657 hybrid plasmids had an additional IncR replicon.

**Figure 1:**
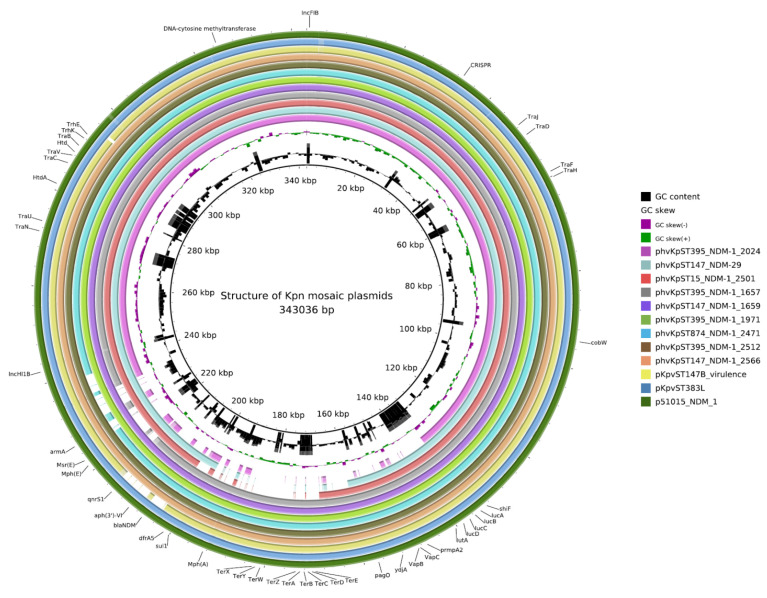
Structure of *K. pneumoniae* mosaic plasmids. Circles from inside to outside show the coding sequence region of phvKpST395_NDM-1_2024, phvKpST147_NDM-29, phvKpST15_NDM-1_2501, phvKpST395_NDM-1_1657, phvKpST147_NDM-1_1659, phvKpST395_NDM-1_1971 (reference), phvKpST874_NDM-1_2471, phvKpST395_NDM-1_2512, phvKpST147_NDM-1_2566, pKpvST147B_virulence, pKpvST383L, and p51015_NDM_1. GC skew (dark green and magenta); GC content (black) is represented in the inner circles. AMR genes, virulence factors, transconjugation, genes of restriction-modification (RM), toxin-antitoxin (TA) systems, and CRISPR array are indicated on the outer circle.

The closest matching plasmids from a global database were pKpvST383L (GenBank accession no. CP034201.2) and pKpvST147B_virulence (GenBank accession no. CP040726.1) of the Kpn strain from two different London hospitals [12], as well as p51015_NDM_1 (GenBank accession no. CP050380.1) from the Czech Republic. The plasmids content, as revealed by whole-genome sequencing (WGS), is shown in Supplementary Material Table S2. Virulence plasmids of 1970_kpn, 2024_kpn, and 2501_kpn were found to be shorter (~315 kb), mostly because they lacked the tellurium resistance cassette in comparison to plasmids in the other hvKp. A schematic representation of the circular genome maps of mosaic plasmids of CR-hvKp Kpn and linear alignment are shown in Figure 1 and Supplementary Materials, Figure S1a, respectively.

Some of the isolates also contained IncFIB/IncFII (2471_kpn, 2501_kpn, 51015), IncR (1659, 1970, 1971), IncL (KpvST383_NDM_OXA-48), and IncFIB (KpvST147B_SE1_1_NDM) plasmids carrying antibiotics and heavy metal resistance genes. 

### 2.4. AMR Determinants

CR-hvKp isolates, together with those described in the previous section, were found to possess an array of AMR genes. In addition to the highly prevalent chromosomal quinoxaline resistance (*oqxAB*) and fosfomycin resistance genes (*fosA*), the chromosome of the KpvST383_NDM_OXA-48 strain also contained *catA1*, *tet(A)*, *mph(A)*, and the chromosome of 2501_kpn contained *aac(3)-IId*. Apart from the *bla*_SHV_ [13], *bla*_CTXM-15_ was found on the ISEcp1-*bla*_CTX-M-15_ element. Class A extended-spectrum beta-lactamases were located on different mobile genetic elements (MGEs) harbored through fusion (IncFIB/IncHI1B, IncFIB/IncFII) or IncR backbone plasmids. *bla*_OXA-9_ and *bla*_TEM-1A_ were prevalent in either Tn6238- or IS26-dependent translocatable units integrated into IncR or IncHI1B/IncFIB plasmid types, or class 1 integrons. *bla*_OXA-48_ was associated with inverted Tn1999.2 on the IncL plasmid, *bla*_OXA-244_, in turn, with IS1R-mediated MGE. *bla*_OXA-1_ together with *catB3* and *aac(6’)-Ib-cr* were linked to IS26-mediated insertion sequences positioned on fusion or IncR backbone plasmids. A variety of MGEs containing *bla*_TEM-1b_ were integrated into the latter types of plasmids in the same manner.

All the isolates were found to carry *bla*_NDM-1_ except for 2024_kpn, which did not contain any MBL genes. In most of the isolates, *bla*_NDM_ was integrated into the IncFIB/IncHI1B plasmid, while in the KpvST147B_SE1_1_NDM isolate, KpvST383_NDM_OXA-48 carried *bla*_NDM-5_. In addition, WGS revealed *bla*_NDM-29_ in 1970_kpn, which was carried on a 311-kb mosaic plasmid referred to as phvKpST147_NDM-29.

### 2.5. bla_NDM_

The new *bla*_NDM-29_ variant differed from *bla*_NDM-1_ by a single amino acid (D130N, 388(G→A)). The mutation probability at the 388th nucleotide of *bla*_NDM_ was detected in 4% of 244 Kpn plasmid sequences containing *bla*_NDM_ (available at GenBank). Similar amino acids substitutions were present in NDM-7 and NDM-19. However, these enzymes carried additional amino acids substitutions: M154L in NDM-7; M154L + A233V in NDM-19. A different substitution at the 130th position presented in NDM-14 (D130G) and NDM-8 (D130G, M154L). NDM-4 with M154L, a double mutant NDM-15 with M154L + A233V, and NDM-6 with A233V were added to the full list of all possible amino acid variations (Table 2).

*bla*_NDM-29_ was located on a Tn125-like hypothetical transposon (Supplementary Material Figure S1b) [14]. In phvKpST147_NDM-29, *bla*_NDM-29_ was flanked upstream by ISAba125-ISspu2-ISAba125 [15,16,17,18] and downstream by IS26, turning into an IS26-dependent composite transposon structure. Additionally, aphA6 was part of the remnant of a composite transposon originally with two terminal direct copies of ISAba14 [19]. Downstream of *bla*_NDM-29,_ coding for *ble-iso-tat-dct*, was truncated at *dct* via the insertion of IS26. IS26s were found at different positions in the NDM genetic environment, which indicated increased activity and multiple independent acquisitions of IS26 [20]. However, as part of the IS26-dependent composite transposon, IS26 played a key role in the mobilization of composite transposons between different plasmids or chromosomes [14]. This genetic context was identical to that of NDM-containing plasmids in the Czech Republic and the UK.

### 2.6. Functional Analysis of bla_ndm-29_

The susceptibility test results showed that the clinical Kpn isolate, 1970_kpn, exhibited high-level resistance to all examined carbapenems, cephalosporins, aminoglycosides, fluoroquinolones, and a piperacillin/tazobactam combination, but was susceptible to aztreonam. The antimicrobial agents and phenotypes of all the tested isolates are listed in Supplementary Materials, Table S4. Antimicrobial susceptibility test results initially suggested the presence of an MBL, which was confirmed by PCR targeting NDM-like sequences.

The expression of *bla*_NDM-29_ and *bla*_NDM-1_ in *E. coli* XL10-Gold, compared to the control strain, resulted in reduced susceptibility to third- and fourth-generation cephalosporins, carbapenems, and piperacillin/tazobactam. All the transformants were susceptible to aztreonam. Both pEGFP-N3 transformants exhibited significantly higher resistance to gentamicin compared to the pJET1.2 transformants, which proved that the first vector contained an aminoglycoside resistance gene.

Under conditions designed to mimic zinc(II) scarcity by including the metal chelator ethylenediaminetetraacetic acid (EDTA), all tested isolates showed a significant decrease in minimal inhibitory concentration (MIC) values for broad-spectrum cephalosporins and carbapenems. Transformants containing NDM-29 showed the same susceptibility to third and fourth generation cephalosporins and carbapenems as NDM-1. Both pJET1.2 transformants demonstrated significantly higher resistance to ampicillin compared to the pEGFP-N3 transformants, even under zinc-limiting conditions, which is a selective marker for ampicillin resistance.

A different substitution at the 130th position presented in NDM-14 (D130G) and NDM-8 (D130G, M154L). Based on our findings, the evaluation of the impact of the D130N substitution on the hydrolytic activity toward β-lactam antibiotics revealed that transformants of *bla*_NDM-29_ exhibited the same β-lactam resistance as the transformants of *bla*_NDM-1_, unlike D130G which has been found to confer higher resistance to meropenem and imipenem than *bla*_NDM-1_ and indirectly influence on the active site [21]. The stability changes at the 130th position (∆∆Gu = −0.3330) is neutral in comparison with NDM-1 according to EASE-MM. The comparison of NDM-1 and NDM-29 structures is represented in Supplementary Materials, Figure S2. 

In contrast, for NDM-7 and NDM-4, the leucine residue at position 154 (M154L) extended the hydrolytic activity toward carbapenems [22,23] and for NDM-4 and NDM-15, the metal-binding [7]. Which is in agreement with [8] showing resistance >3–4 dilution increases in MIC values for two or more antibiotics in zinc-depleted condition of NDM-4, 8, 7, 15. A233V, in turn, was not only a stabilizing substitution, not affecting zinc-binding abilities [7], but also appeared to further increase the carbapenemase activity under zinc-limiting growth conditions [24]. The D130N amino acid change also presented in NDM-29, 7, 19. We suggest that the asparagine residue at the 130th position does not have an effect on the formation of the active site.

### 2.7. Virulence

The hybrid plasmids of the analyzed bugs contained key virulence determinants, such as aerobactin cluster (*iucABCD*) and its receptor (*iutA*), capsule upregulation (*_p_rmpA*, *_p_rmpA2*), metabolite transporters (*peg-344, pagO*), other genes associated with hypervirulence (*cobW, luxR, shiF*, and *ydjA*), and genes encoding heavy metal resistance such as *terBEDWXZ* (tellurium). Unlike the rest, 2024_kpn contained the smallest set of virulence genes lacking *iucBCD, iutA, _p_rmpA2, pagO,* and *ydjA*, which were prevalent in the remaining mosaic plasmids. In addition, all of the hybrid plasmids harbored genes coding for conjugal transfer proteins, suggesting conjugative plasmid transfer. The co-occurrence of other heavy metal resistance genes, such as *silABCEFGPRS* (silver), *arsABCDR* (arsenic), and *pcoABCDERS* (copper), was revealed in all IncFIB/IncFII plasmids and *merARCTP* (mercury) in several IncR plasmids.

The key pathogenicity factors, carried as core genes by the chromosomes of the investigated Kpn isolates, were lipopolysaccharide (2-keto-3-deoxy-D-manno-octulosonate-8-phosphate synthase (*kds*), a moiety of lipid A endotoxin), enterobactin (siderophore enterobactin (*ent*), ferric enterobactin-binding periplasmic protein (*fep*)), *E*. *coli* common pilus (*ecp*), and type 1 fimbriae (*fim*) genes. Yersiniabactin synthesis (*ybt* locus) was prevalent in half of the analyzed genomes, including all the representatives of ST395. Silver resistance gene (*silA*) was found on the chromosome of 1971_kpn, 2512_kpn, KpvST147B_SE1_1_NDM, and KpvST383_NDM_OXA-48 strains. In KpvST147B_SE1_1_NDM, the *ecp* locus was mobilized by putative integrative and mobilizable elements (IME). In Kpn 51015 and 2512_kpn strains, an additional siderophore system (*ybt*), its receptor locus (*fyu*), and iron regulatory protein (*irp*) genes were expressed by putative ICE with T4SS, which was integrated into the chromosome.

Silver resistance gene (*silA*), usually located on the virulence plasmids, was found on the chromosome of 1971_kpn, 2512_kpn, KpvST147B_SE1_1_NDM, and KpvST383_NDM_OXA-48 strains. In KpvST147B_SE1_1_NDM, the *ecp* locus was mobilized by putative IME. In Kpn 51015 and 2512_kpn strains, an additional siderophore system (*ybt*), its receptor locus (*fyu*), and iron regulatory protein (*irp*) genes, acquired by putative ICE with T4SS, were integrated into the chromosome.

### 2.8. Restriction Modification and TA Systems

The type II methyltransferase M.Kpn34618Dcm was present on the chromosomes of the analyzed isolates. The chromosomes of 1659_kpn and 2566_kpn isolates carried the type I restriction enzymes StySKI and 1970_kpn M.Sen1921I methyltransferase (type I). As for the mosaic plasmids, a DNA cytosine methyltransferase was found in all isolates except for 2024_kpn.

Using a collection of Pfam toxin profiles embedded in SLING, we identified 21 toxin groups (18 of type II and three of type IV (CbtA, Cpta, AbiEii)). TA systems were highly prevalent in all isolates of Kpn, with a median of 17 loci per genome. GNAT_acetyltran, HD, HipA, Cpta, and Fic toxin domains were highly prevalent on the chromosomes of the analyzed Kpn isolates. Different combinations of HigB, CcdB, CbtA, ParE, PIN, Gp49, RelE, Polyketide_cyc2, YdaT, PIN, and AbiEii toxin domains were also found.

Equally important is the *vapBC* of the type II TA system that has been identified on all mosaic plasmids. In addition, higAB (PIN domain) was integrated into the hybrid plasmids of 1657_kpn and 2512_kpn. PIN, ParE, RelE, Gp49 were also observed on different plasmids besides mosaic ones together with HicA, NTP_transf_2, PemK, YoeB, CbtA, and ANT toxin domains.

### 2.9. CRISPR-Cas System

Although the prevalence of IncF plasmids was recognized among strains in the absence of CRISPR-Cas systems [25], type I-E CRISPR-Cas locus with one CRISPR array was identified in the chromosomes of 1659_kpn, 1970_kpn, and 2566_kpn, and type I-E* CRISPR system with one CRISPR array in 2501_kpn. It consisted of eight genes (cas1,2,3,5, cse1,2,3,4) of the *cas* operon and one CRISPR array with 43 identical spacers, and the mosaic plasmids of all the analyzed isolates did not contain protospacers. We found the occurrence of a type I-E* CRISPR system with two identical CRISPR arrays (10 + 9 spacers) in KpvST147B_SE1_1_NDM and 5105 strain chromosomes. The isolate KpvST383_NDM_OXA-48 harbored the type I-E CRISPR-Cas locus with one CRISPR array consisting of 15 spacers, the last of which coincided with the last spacer of the CRISPR array with 43 spacers.

Similarly, a CRISPR array with 16 spacers was found on the aforementioned mosaic plasmids not adjacent to *cas* genes (12 spacers on phvKpST395_2024). One spacer of all the mosaic plasmid CRISPR arrays contained a Gifsy-2 prophage sequence located on the chromosomes of all the aforementioned strains (except for KpvST147B_SE1_1_NDM and 5105). Similarly, the hybrid plasmids carried another spacer that matched the traL of the Kpn conjugative IncF plasmid. This ensured the barrier for acquiring the IncF mosaic plasmid, which prevented the decrease in fitness due to the probable presence of multiple AMR plasmids.

## 3. Discussion

Here, we showed the emergence of Kpn isolates of different STs (ST15, ST147, ST395, and ST874) with hybrid plasmids coharboring virulence genes and *bla*_NDM_ in the hospitals in St. Petersburg. 

Alignment of four distinct plasmid types, phvKpST147_NDM-29, phvKpST395_2024, and pKpvST147B_virulence showed the diversification of plasmid branches (Supplementary Materials, Figure S1d). We suggest the existence of a progenitor of the phvKpST395_2024 plasmid of the IncFIB/IncHI1B replicon type with a full hypervirulence gene cluster (predecessor 1). Possible evolutionary pathways that generated four distinct types of mosaic plasmids were as follows: (1) the acquisition of Tn-125-like (such as study isolates 1970_kpn, 1971_kpn, 2501_kpn) or (2) tellurium resistance gene cluster (such as pKpvST147B_virulence in KpvST147B_SE1_1_NDM strain) by predecessor 2 following the acquisition of the region containing Tn1548 by predecessor 1; (3) an intermediate type of mosaic plasmid produced by the acquisition of Tn125-like genes from the IncFIB plasmid by the type 2 plasmid, as seen in Supplementary Materials, Figure S1c (such as study isolates 1659_kpn, 2471_kpn, 2512_kpn, 2566_kpn); (4) deletion of the region containing genes associated with hypervirulence (*iutA, iucBCD, _p_rmpA2, cobW, pagO, ydjA*) and fusion with the IncR plasmid (phvKpST395_2024 in such as the isolate 2024_kpn). A schematic representation of the possible evolutionary pathway is shown in Supplementary Materials, Figure S3.

We speculated that, given the simultaneous presence of pKpvST147B_virulence, carrying the tellurium resistance cluster, and pKpvST147B_NDM_1 carrying a Tn125-like sequence within KpvST147B_SE1_1_NDM in the UK, this strain was the predecessor of the convergence of Te resistance and Tn125 in one vector, found in phvKpST395_NDM-1_1657, phvKpST147_NDM-1_1659, phvKpST874_NDM-1_2471, phvKpST395_NDM-1_2512, and phvKpST147_NDM-1_2566. The linear alignment of phvKpST147_NDM-29, pKpvST147B_virulence, and pKpvST147B_NDM_1 is shown in Supplementary Materials, Figure S1c. These perfect mosaic structure hybrid plasmids converging MDR and hv biphenotypes within a single vector are a shortcut to the evolutionary success of these superbugs.

Our data are consistent with the previously published hypothesis for the emergence of hybrid plasmids stating the acquisition of resistance genes by a virulent plasmid [4].

Because of recombination following transmission, convergent plasmids, which simultaneously enable virulence and resistance, and have enhanced genetic plasticity, were generated. All NDM-producing CR-hvKp isolates were from multiple STs and different geographical regions, suggesting the convergence of the resistance and virulence genes in conjugative plasmids. Mosaic structures were formed upon the fusion of carbapenemase-encoding genes and virulence plasmids via MGEs [4]. Despite the high identity of the analyzed mosaic plasmids, each particular lineage of Kpn was associated with a different mobilome, suggesting an IS-mediated combination that results in such hybrid plasmids.

TA systems have not been studied well in the Kpn species complex. Overall, TA systems are associated with the stabilization of MGE-containing virulence genes and/or AMR genes and maintenance of plasmids via postsegregational killing [26]. Our data are in agreement with previous findings of high prevalence of II TA systems among virulence plasmids [27]. VapC toxin containing a pilus retraction protein (PilT) N-terminal (PIN domain) acts as a ribonuclease that cleaves RNA molecules, thereby reducing the rate of translation, and *vapB* encodes a matching antitoxin. The role of *vapBC* is related to bacterial pathogenicity [28]. HigB is a ribosome-dependent mRNA endoribonuclease that inhibits cell proliferation, whereas the *higA* antitoxin prevents the cessation of cell growth.

In the present study, we also identified CRISPR array sequences. A possible role of the CRISPR-Cas system on the mosaic plasmid is to contribute to the homologous recombination of AMR and virulence determinants in a single vector and probable promotion of cointegration plasmid formation [29] because host plasmids are fused per se.

In light of the high sequence identity of the isolated array repeats on the mosaic plasmids to the repeats in the complete CRISPR-Cas systems of closely related genomes, and the absence of transposable elements in the vicinity of isolated arrays, the most probable route of the evolution of the isolated CRISPR array is the loss of *cas* genes in a CRISPR-Cas locus [30]. It remains unclear whether the array isolated in this study is functional; however, it is known to be active through trans utilization by Cas proteins. Similarly, it is not apparent why CRISPR positive isolates did not recognize IncF plasmid as a foreign DNA.

Moreover, in one of the isolates, *bla*_NDM_ was identified as a new variant (*bla*_NDM-29_). Contrary to what we found here, in another study, D130N added stability against degradation and tolerance to Zn scarcity in NDM-7, unlike NDM-4 [7]. D130N suggested the formation of a separate branch in the evolution of NDM enzymes. The evolution of β-lactamases is usually aimed at a gradual increase in the AMR. On the other hand, evolutionary paths may not lead to the formation of highly effective branches and can be the result of a random mutation. In this case, we cannot be sure whether the mutation at position 130 was a step in the evolutionary process or an independent event. In addition, amino acids presented in NDM-29 were not located in what has been identified as the active site of NDM enzyme or the amino acid residue that binds to the zinc ions (His120, His122, His189, His250, Cys208, and Asp124) [31], despite its close occurrence.

## 4. Materials and Methods

### 4.1. Identification of Bacterial Strains and Antimicrobial Susceptibility Testing

Identification of bacterial species was performed using MALDI-TOF MS (Bruker Daltonik, Bremen, Germany). The string test [32] was used for the detection of hypermucoviscosity and PCR for the detection of carbapenemase genes [33]. The MICs of ampicillin, cefotaxime, cefoxitin, ceftriaxone, ceftazidime, cefepime, aztreonam, meropenem, imipenem, ertapenem, biapenem, gentamicin, amikacin, ciprofloxacin, tigecycline, fosfomycin, trimethoprim-sulfamethoxazole, ceftazidime-avibactam, and aztreonam–avibactamand piperacillin/tazobactam were determined according to ISO 20776-1 (2006). Determination of the MICs of β-lactams under zinc-limiting conditions (50 μM EDTA) was conducted according to CLSI recommendations (M100 ED30:2020).

### 4.2. Cloning

*bla*_NDM-29_ and *bla*_NDM-1_ cloning was conducted as described in Supplementary Material Text S1.

### 4.3. Genomic Sequencing

Nine isolates were characterized via Illumina MiSeq sequencing (Nextera XT libraries, paired-end 300-bp reads), and reads were assembled de novo into contigs using the SPAdes algorithm v.3.10.1. Only contigs longer than 5 kb were analyzed. Long reads were obtained using an Oxford Nanopore MinION Sequencer (SQK-LSK109 and flow cell R9.4.1). MinION reads were based on the Guppy software (v4.0.11) available from Oxford Nanopore technologies. The de novo hybrid assembly of both short Illumina reads, and long MinION reads were performed using Unicycler (v0.4.7).

### 4.4. Sequence Analysis

The isolates were screened for AMR genes, STs, plasmid replicon types, and RM systems at the Center for Genomic Epidemiology (http://www.genomicepidemiology.org, accessed on 2 February 2021), and virulence genes using ABRicate (www.github.com/tseemann/abricate, accessed on 2 February 2021). The presence of virulence factors was confirmed using the virulence database at the Pasteur Institute for Kpn (www.bigsdb.pasteur.fr, accessed on 2 February 2021). K and O antigen loci were determined using Kaptive [34]. The genomes were functionally annotated using the PATRIC platform (accessed on 24 July 2020). The presence of five genetic markers of the hv phenotype (*peg-344, iroB, iucA, _p_rmpA*, and *_p_rmpA2*) was verified using previously published primers [35]. Polymorphism analysis was performed using Riddikulus (www.github.com/dariader/Riddikulus, accessed on 5 March 2021). CRISPR array sequences were identified using CRISPRFinder (www.crispr.i2bc.paris-saclay.fr/Server/, accessed on 2 February 2021). Spacers from CRISPR arrays were analyzed for their identity in GenBank via nucleotide BLAST search. SLING (2.0.1) was used to search for toxins and their cognate antitoxins using the built-in toxin domain database [36]. Each IS element was classified according to the ISFinder database (accessed on 2 February 2021) [37]. ICEs were detected by ICEfinder (www.db-mml.sjtu.edu.cn/ICEfinder/ICEfinder.html, accessed on 2 February 2021). The circular plasmids were visualized using BRIG (v.0.95) [38], and linear alignment was performed using Easyfig (v.2.2.2) [39].

The virulence and AMR score were calculated as shown previously [40], which reflects the accumulation of loci associated with the increased risk of clinically relevant AMR or hypervirulence.

The contig with the new *bla*_NDM-29_ was deposited in GenBank (accession number MN624980; GenBank BioProject accession number PRJNA522420) as well as mosaic plasmids: phvKpST395_NDM-1_1657 (accession number CP072809; GenBank BioProject accession number PRJNA719704), phvKpST147_NDM-1_1659 (accession number CP072810; GenBank BioProject accession number PRJNA719707), phvKpST147_NDM-29 (accession number CP066856; GenBank BioProject accession number PRJNA522420), phvKpST395_NDM-1_1971 (accession number MW911666), phvKpST395_NDM-1_2024 (accession number MW911667), phvKpST874_NDM-1_2471 (accession number MW911668), phvKpST15_NDM-1_2501 (accession number MW911669), phvKpST395_NDM-1_2512 (accession number MW911670), phvKpST147_NDM-1_2566 (accession number MW911671). NDM-29 protein stability was calculated on EASE-MM server [41]. Protein crystal structures were derived from PDB (www.rcsb.org/; accessed on 3 May 2021).

### 4.5. MURINE Model of Sepsis

The virulence of eight isolates was assessed via a mouse lethality assay, and LD_50_ values were determined as described previously [9].

## 5. Conclusions

The discovery of structurally similar plasmids in geographically distant regions suggests that the actual distribution of hybrid plasmids carrying virulence and resistance genes is much wider than expected. The convergence of virulence and AMR determinants in a single vector, together with the rapid evolution of NDMs, poses a global threat to healthcare systems.

## Figures and Tables

**Table 1:** Characteristics of patients and CR-hvKp isolates.

Isolate	Year of Isolation	Location	ST	Capsule Type	Mouse Lethality (LD_50_, CFU)	*bla*_NDM_	Virulence Scores	Resistance Score	Predominant Diagnosis	Outcomes
1657_kpn	2017	SPb_1 ^1^	395	KL2	10^2^	*bla*_NDM-1_	4	2	Adenocarcinoma of the sigmoid colon Breast cancer	death
1659_kpn	2017	SPb_1	147	KL20	10^4^	*bla*_NDM-1_	3	2		
1971_kpn	2018	SPb_1	395	KL2	10^5^	*bla*_NDM-1_	4	2	Pancreatic cancer	no change
1970_kpn	2018	SPb_1	147	KL20	10^4^	*bla*_NDM-29_	3	2	Prostate cancer	improvement
2024_kpn	2018	SPb_1	395	KL2	10^5^	-	1	2	B cell lymphoma	no change
2471_kpn	2019	SPb_1	874	KL45	10^4^	*bla*_NDM-1_	3	2	Hysterocarcinoma, IA st., pT1aN0M0	no change
2501_kpn	2019	SPb_1	15	KL19	10^4^	*bla*_NDM-1_	3	2	Rectal cancer cT3N1M0, IV Stomach body cancer T2N1M0	improvement
2512_kpn	2019	SPb_2 ^2^	395	KL2	10^3^	*bla*_NDM-1_	4	2	Cornea injury	left eye vision loss
2566_kpn	2019	SPb_1	147	KL20	10^4^	*bla*_NDM-1_	3	2	Follicular lymphoma, grade 1–2, IV	improvement

^1^ SPb_1—Russia, oncology center, in Saint Petersburg; ^2^ SPb_2—Russia, general hospital, in Saint Petersburg.

**Table 2:** *bla*_NDM_ enzymes with similar amino acids.

Enzymes	Amino Acids		
*bla*_NDM-1_	-	-	-
*bla*_NDM-29_	N130	-	-
*bla*_NDM-__14_	G130	-	-
*bla*_NDM-4_	-	L154	-
*bla*_NDM-6_	-	-	V233
*bla*_NDM-__8_	G130	L154	-
*bla*_NDM-7_	N130	L154	-
*bla*_NDM-15_	-	L154	V233
*bla*_NDM-19_	N130	L154	V233

## Supplementary Materials

The following are available online at https://www.mdpi.com/article/10.3390/antibiotics10060691/s1. Table S1: The clinical details of the patients from which study isolates of Kpn were collected; Table S2: Phenotypic and genotypic characteristics of twelve CR-hvKP (1657_kpn, 1659_kpn, 1970_kpn, 1971_kpn, 2024_kpn, 2471_kpn, 2501_kpn, 2512_kpn, and 2566_kpn) obtained using hybrid genome assembly, in comparison with the previously reported isolates with hybrid plasmids KpvST147B_SE1_1_NDM, KpvST383_NDM_OXA-48, 51015; Table S3: Results of susceptibility testing of Kpn isolates (MIC, mg/L); Table S4: MICs for the NDM variants under standard and zinc-limiting conditions (50 μM EDTA). Shown are the MICs (mg/L) for β-lactams of the clinical isolate 1970_kpn of Kpn, *E. coli* XL10-Gold, *E. coli* XL10-Gold harboring *bla*_NDM-1_ and *bla*_NDM-29_; Figure S1: (a) Linear alignment of mosaic plasmids: phvKpST395_NDM-1_2024, phvKpST395_NDM-1_1657, phvKpST147_NDM-1_1659, phvKpST147_NDM-29, phvKpST395_NDM-1_1971, phvKpST874_NDM-1_2471, phvKpST15_NDM-1_2501, phvKpST395_NDM-1_2512, phvKpST147_NDM-1_2566, pKpvST147B_virulence, pKpvST383L, and p51015_NDM_1; (b) Description of gene clusters; (c) Genome comparison of pKpvST147B_NDM_1, phvKpST147_NDM-29, phvKpST147_NDM-1_2566 and pKpvST147B_virulence; (d) Genome comparison of phvKpST395_NDM-1_2024, phvKpST147_NDM-29, and pKpvST147B_virulence; Figure S2: The structure of NDM-1 and NDM-29; Figure S3: Possible evolutionary pathways that generated four distinct types of mosaic plasmids; Text S1: Cloning of *bla*_NDM-29_ and *bla*_NDM-1_.
