# Supplementary material for: Emergence of Hybrid Resistance and Virulence Plasmids Harboring New Delhi Metallo-β-Lactamase in Klebsiella pneumoniae in Russia
Source: Antibiotics (Basel). 2021 Jun 9;10(6):691. doi: 10.3390/antibiotics10060691 (PMC8226487; doi:10.3390/antibiotics10060691)
Supplement: Supplementary file 1 [file antibiotics-10-00691-s001.zip › Supplementary Material Fig S1.pdf]

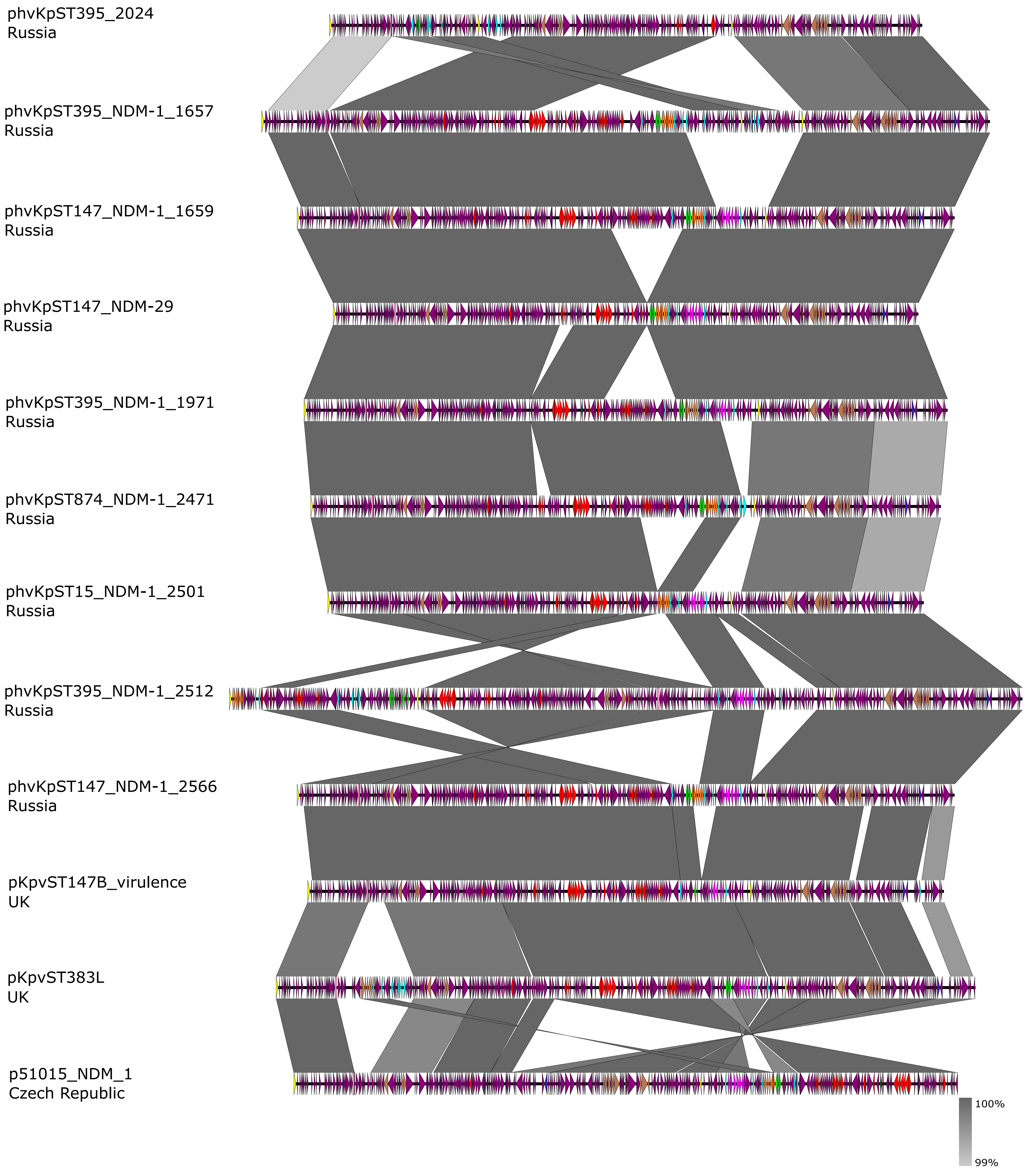

Figure S1a: Linear alignment of mosaic plasmids: phvKpST395_2024, phvKpST395_NDM-1_1657, phvKpST147_NDM-1_1659, phvKpST147_NDM-29, phvKpST395_NDM-1_1971, phvKpST874_NDM-1_2471, phvKpST15_NDM-1_2501, phvKpST395_NDM-1_2512, phvKpST147_NDM-1_2566, pKpvST147B_virulence, pKpvST383L, and p51015_NDM_1.

Plasmids are shown starting from IncFIB replication. Description of gene clusters is represented on Figure S1b.
protein gene. Homologous regions are shaded dark grey. Genes are indicated in colors as follows:
- capsule up-regulation genes ($_{p}$rmpA, $_{p}$rmpA2), aerobactin cluster genes (iucABCD, iutA), genes associated with hypervirulence (terABCDEWXYZ - tellurium resistance proteins, pagO - which protects bacteria from phagocytosis, cobW - involved in cobalamin biosynthesis, luxR - coding for a quorum-sensing regulator controlling virulence gene expression, shiF - promoting transfer of lysine, ydjA - nitroreductases), marked red arrows;
- resistance genes (aph(3')-I, III, VI - aminoglycoside 3'-phosphotransferase, qnrS1 - quinolone resistance protein, sul2 - dihydropteroate synthase type 2, mph(A) - macrolide resistance, catA1 - chloramphenicol O-acetyltransferase, aac(6')-Ib - aminoglycoside N(6')-acetyltransferase, aadA1 - aminoglycoside 3''- nucleotidyltransferase, *bla*$_{\text{OXA-1, 9, 48}}$ - class D β-lactamase, *bla*$_{\text{CTX-M-15}}$, *bla*$_{\text{TEM-1A,B}}$ - class A β-lactamase), cian;
- genes of Tn125-like, orange;
- genes of integron class 1, green;
- genes of Tn1548, marked lilac;
- replication initiation protein genes, yellow;
- genes encoding various conjugal transfer proteins (IncF traBCDFHJNUV, IncH1 trhAEKT, orf4), brown;
- genes associated with RM systems, dark blue;
- CRISPR arrays, pink rectangulars;
- genes associated with TA systems, dark green arrows;
- other accessory genes, purple.

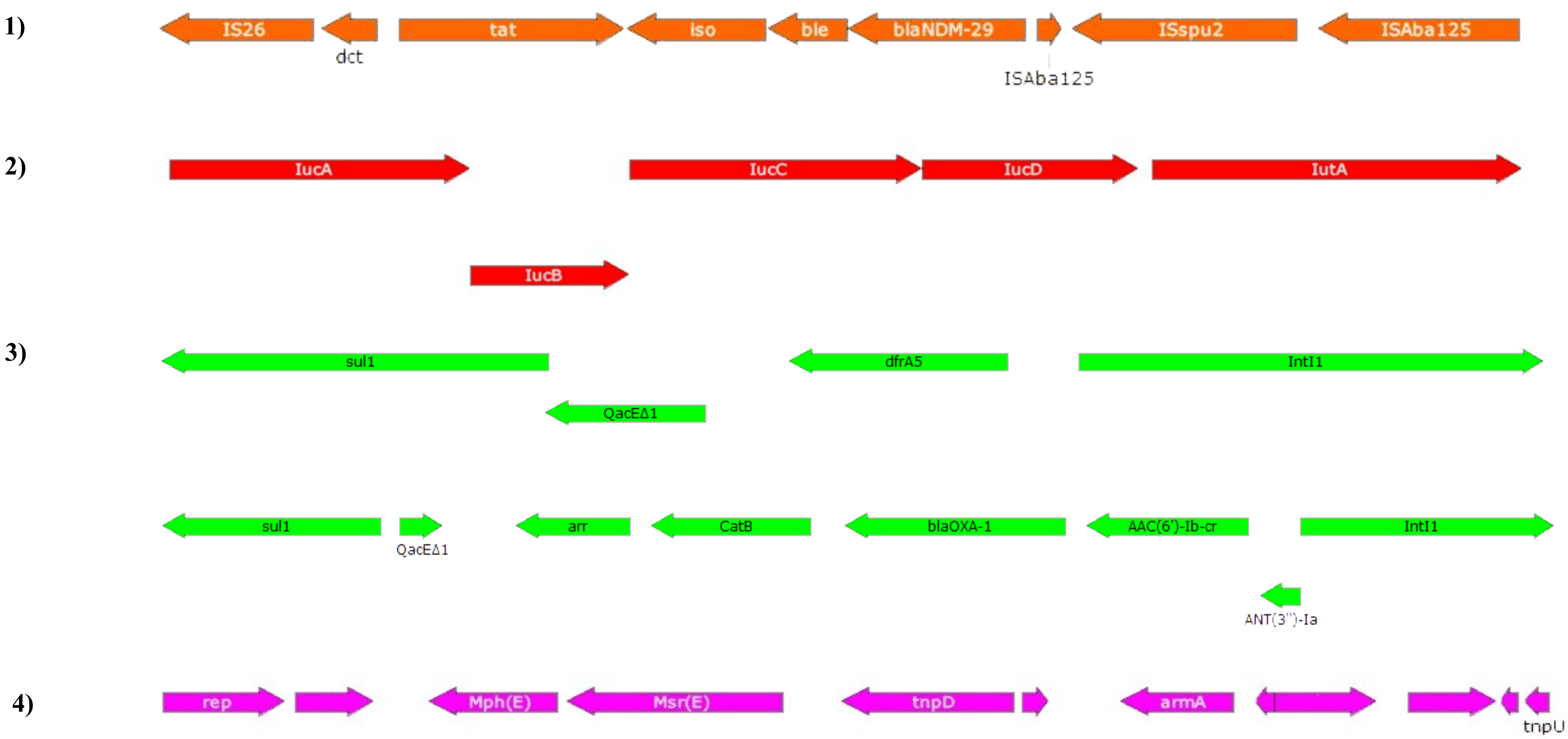

Figure S1b: Description of gene clusters.
(1) Genes of Tn125-like (IS26, dct - divalent cation tolerance, tat - oxidoreductase, iso - phosphoribosylanthranilate isomerase, ble - bleomycin resistance protein, bla_{NDM} - MBL β-lactamase, ISAba125, ISspu2) marked orange arrows.
(2) Organization of aerobactin cluster genes (iucA - aerobactin synthase, iucB aerobactin siderophore biosynthesis protein, iucC - aerobactin synthase, iucD - NADPH-dependent L-lysine N(6)-monooxygenase, iutA - ferric aerobactin receptor) marked red.
(3) Schematic representation of the genes of integron class 1 (sul1 - dihydropteroate synthase type 1, dfrA5 - trimethoprim-resistant dihydrofolate reductase, arr - rifampin ADP-ribosyl transferase, catB - chloramphenicol O-acetyltransferase, $bla_{\mathrm{OXA-1}}$ - class D β-lactamase, aac(6')-Ib-cr - aminoglycoside N(6')-acetyltransferase, ant(3")-Ia - aminoglycoside 3"-nucleotidyltransferase, intI1 – class 1 integrase) marked green. Integron class 1 of pKpvST147B_NDM_1 plasmid is represented at the bottom.
(4) Organization of the part of Tn1548 structure (rep - replicase gene, mph(E) - macrolide 2'-phosphotransferase, msr(E) - macrolide efflux protein, tnpU, tnpD - transposon-related genes, armA - aminoglycoside resistance methylase).
The images are derived from Snapgene, gene-editing software.

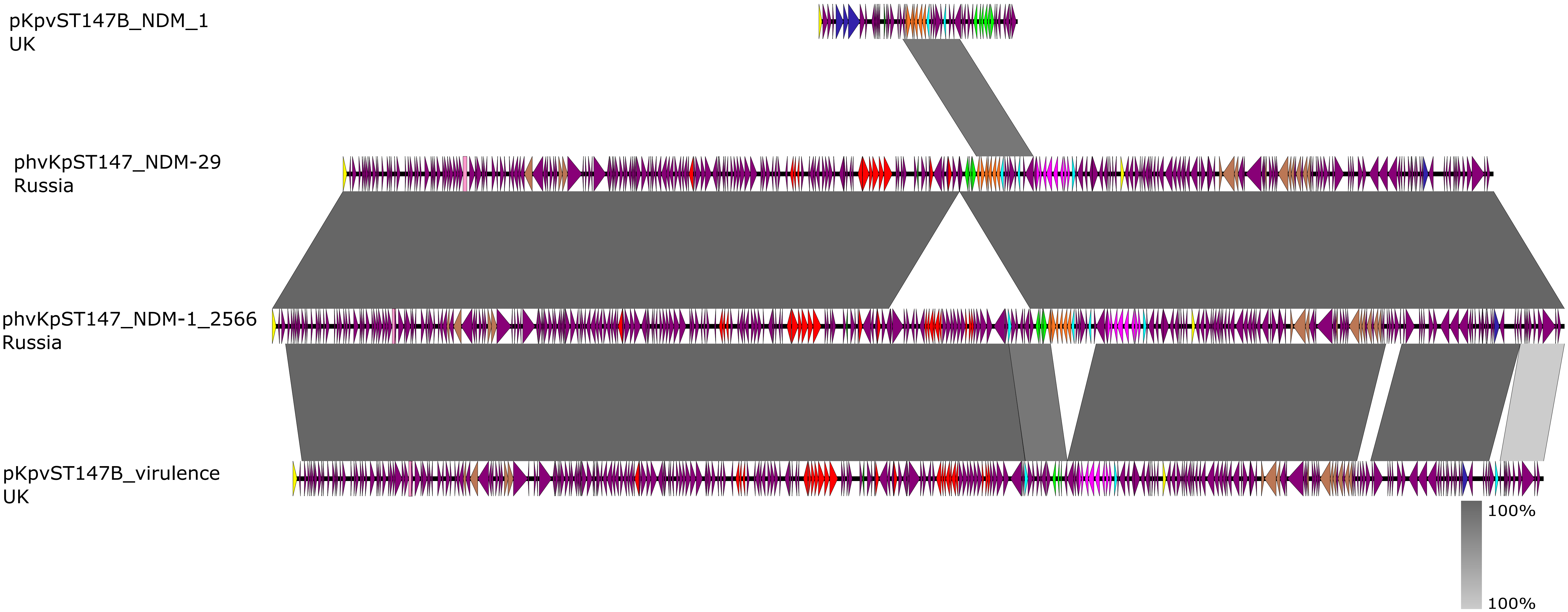

Figure S1c: Genome comparison of pKpvST147B_NDM_1, phvKpST147_NDM-29, phvKpST147_NDM-1_2566 and pKpvST147B_virulence. Colours correspond to Figure S5a,b.

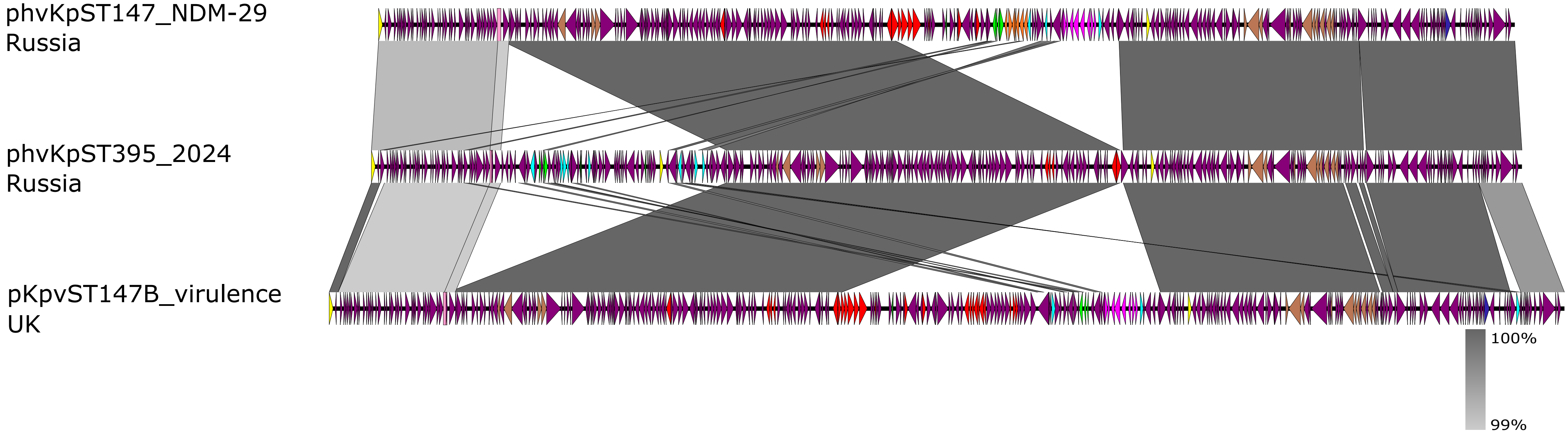

Figure S1d: Genome comparison of phvKpST395_2024, phvKpST147_NDM-29, and pKpvST147B_virulence. Colours correspond to Figure S5a,b.
