# Supplementary material for: Emergence of Hybrid Resistance and Virulence Plasmids Harboring New Delhi Metallo-β-Lactamase in Klebsiella pneumoniae in Russia
Source: Antibiotics (Basel). 2021 Jun 9;10(6):691. doi: 10.3390/antibiotics10060691 (PMC8226487; doi:10.3390/antibiotics10060691)
Supplement: Supplementary file 1 [file antibiotics-10-00691-s001.zip › Supplementary Material Fig S2.pdf]

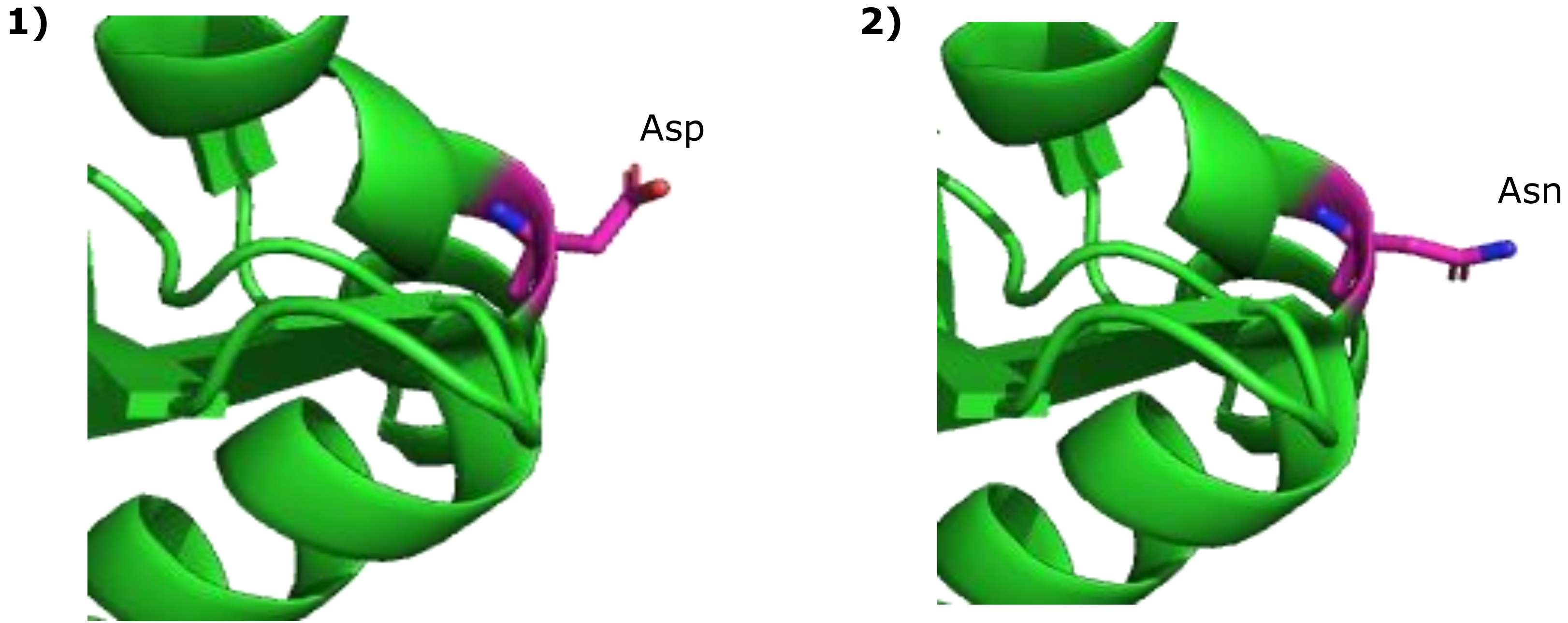

Figure S2: (1) The structure of *bla*$_{\text{NDM-1}}$, where the aspartic acid at 130 position displayed as lilac sticks (PDB entry 5ZR8). (2) The structure of *bla*$_{\text{NDM-29,}}$ where the asparagine at 130 position is highlighted. The oxygen atoms are colored red and amino groups are colored blue. The figures were produced using PyMOL (Schrödinger LLC).
