# Supplementary material for: Emergence of Hybrid Resistance and Virulence Plasmids Harboring New Delhi Metallo-β-Lactamase in Klebsiella pneumoniae in Russia
Source: Antibiotics (Basel). 2021 Jun 9;10(6):691. doi: 10.3390/antibiotics10060691 (PMC8226487; doi:10.3390/antibiotics10060691)
Supplement: Supplementary file 1 [file antibiotics-10-00691-s001.zip › Supplementary Material Fig S3.pdf]

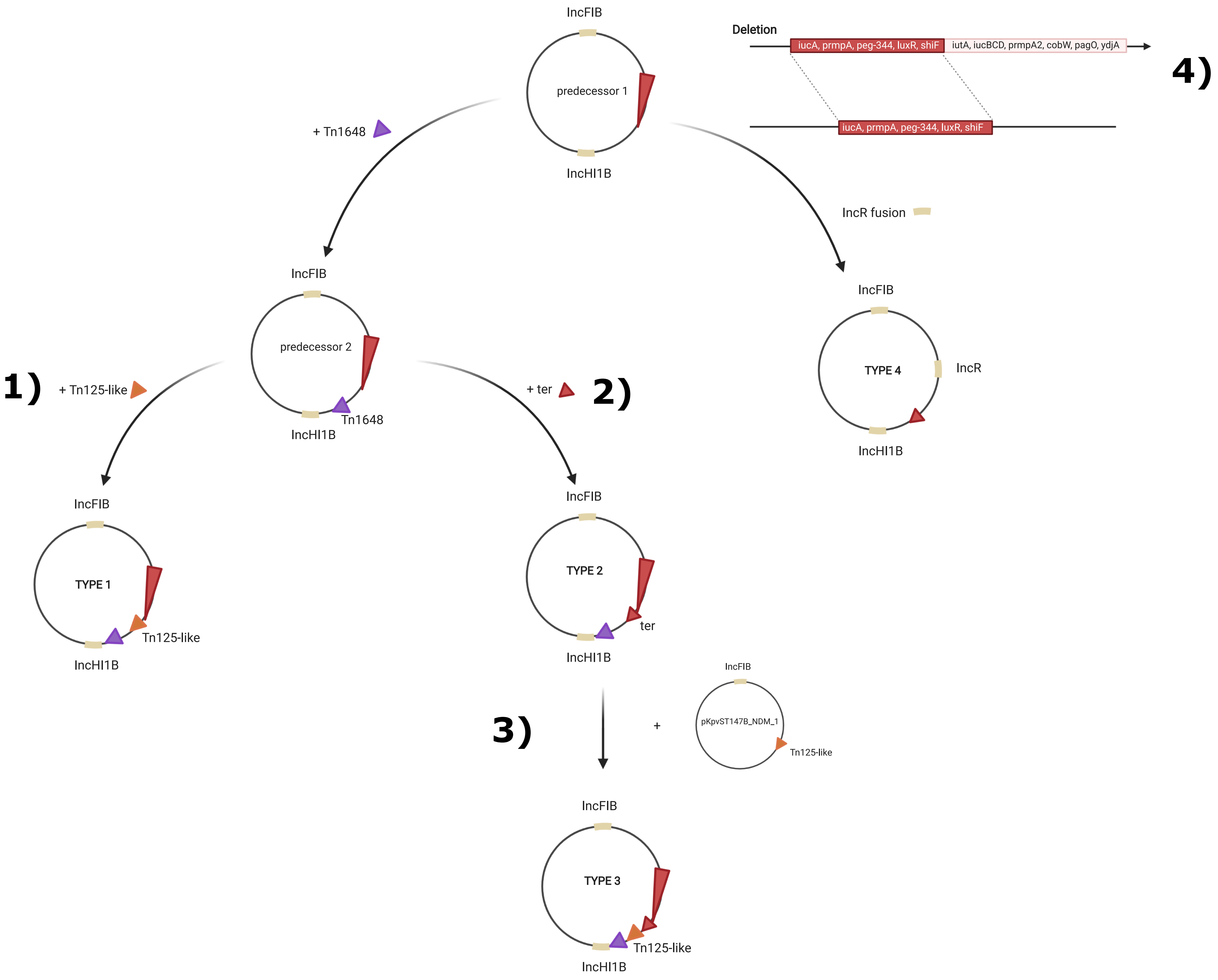

Figure S3: Possible evolutionary pathways that generated 4 distinct types of mosaic plasmids are: (1) the acquisition of Tn-125-like (such as study isolates 1970_kpn, 1971_kpn, 2501_kpn) or (2) tellurium resistance gene cluster (such as pKpvST147B_virulence in KpvST147B_SE1_1_NDM strain) by predecessor 2 following the acquisition by predecessor 1 of region containing Tn1548; (3) acquisition of Tn125-like form IncFIB plasmid by type 2 plasmid (such as study isolates 1659_kpn, 2471_kpn, 2512_kpn, 2566_kpn); (4) deletion the part of the region containing genes associated with hypervirulence (iutA, iucBCD, $_{p}$rmpA2, cobW, pagO, ydjA) and fusion with IncR plasmid (phvKpST395_2024 in such as study isolate 2024_kpn).
