# Supplementary material for: Emergence of Hybrid Resistance and Virulence Plasmids Harboring New Delhi Metallo-β-Lactamase in Klebsiella pneumoniae in Russia
Source: Antibiotics (Basel). 2021 Jun 9;10(6):691. doi: 10.3390/antibiotics10060691 (PMC8226487; doi:10.3390/antibiotics10060691)
Supplement: Supplementary file 1 [file antibiotics-10-00691-s001.zip › Supplementary Material Tab S1.pdf]

Table S1: The clinical details of the patients from which study isolates of Kpn were collected.

Patient No.	Micro No.	Admission date	CR-hvKp isolation date	Sex	Age	Unit [a]	Being in ICU during this hospitalization	Hospital [b]	*bla*$_{NDM}$	Mouse lethality (LD_{50}, CFU)	Predominant diagnosis	Liver metastasis	Infectious complications due to CR-hvKp	Comorbidity [c]	Antibacterials		Surgery	Outcomes	Importation of Klebsiella from another hospital	Prior hospitalization
Patient 1	1659	12.04.2017	27.04.2017	F	unknown	CU	yes	1	NDM-1	10^4	Adenocarcinoma of the sigmoid colon<br>Breast cancer	no	Yes.<br>Infection of the skin and soft tissues<br>Pneumonia<br>Sepsis	CHD<br>AC<br>HD 2st<br>DM<br>Obesity 2st<br>Pyonephrosis<br>Nephrectomy	1)Ciprofloxacin, metronidazole<br>2)Cefoperazone/Sulbactam<br>3) Ciprofloxacin, metronidazole<br>4) Meropenem<br>5) Vancomycin<br>6) Fluconazole<br>7) Imipenem<br>8) Colistin<br>9) Tygecyclin	1) 20.04. (1 day)<br>2) 21.04-23.04. (2 days)<br>3) 23.04. (1 day)<br>4) 24-25.04. (2 days)<br>5) 24.04-27.04. (4 days)<br>6) 25.04-01.05.(7 days)<br>7) 26.04.-01.05. (6 days)<br>8) 27.04-01.05. (5 days)<br>9) 29.04-01.05. (3 days)	yes	Death	no	yes
	1657		26.04.2017						NDM-1	10^2										
Patient 2	1970	26.03.2018	04.04.2018	M	64	DPD	no	1	NDM-29	10^4	Prostate cancer	no	Urinary infection due to epicystostomy.	CHD<br>AC<br>HD 2st<br>MVD<br>AS (<AH 2st)<br>CVD<br>CHF	1) Ciprofloxacin<br>2) Imipenem.	1) 26-28.03.18 (3 days)<br>2)29.03.-01.04.18 (4 days)	no	IIprovement	unknown	no
Patient 3	1971	12.03.2018	05.04.2018	M	unknown	AU	no	1	NDM-1	10^5	Pancreatic cancer	no	Postoperative pancreatitis<br>Pancreatic fistula<br>Abscess in the area of the triangular ligament of the liver and in the lesser omentum<br>Drained common bile duct.	Chronic bronchitis<br>DM	Ciprofloxacin, Metronidazole.	19-21.03.18 (3 days), 28.03-03.04.18 (7 days).	yes	no change	yes	yes
Patient 4	2501	12.07.2019	26.07.2019	M	67	AU	yes	1	NDM-1	10^4	Rectal cancer cT3N1M0, IV<br>Stomach body cancer T2N1M0	yes	Postoperative biloma (after liver resection)<br>Atypical resection of the 3rd and 4th liver segments<br>Biliary peritonitis	CHD<br>AC<br>HD 3 st.<br>DM<br>Condition after left-sided phlebectomy.	1) Ciprofloxacin, Metronidazole<br>2) Amoxicilline/Clavulanic acid 3) Meropenem, Fosfomycin.	1) 17-18.07.19 (2 days)<br>2) 19-25.07.19 (7 days)<br>3) 26.07.-01.08.19(7 days)	yes	Improvement	yes	yes

Patient 5	2566	18.07.2019	31.07.2019	M	49	RU	no	1	NDM-1	10^4	Follicular lymphoma, grade 1-2, IV	no	Sepsis<br>Pyelonephritis<br>Latent mastoiditis<br>Otitis	Urolithiasis.<br>Cholelithiasis.	1) Amoxicilline/Clavulanic acid;	1) 24-29.07.19 (6 days)	yes	Improvement	yes	yes
Patient 6	2471	03.06.2019	17.06.2019	F	60	SBSTU	no	1	NDM-1	10^4	Hysterocarcinoma, IA st., pT1aN0M0	yes	Bacteriuria<br>Ureterohydronephrosis	DM<br>Obesity<br>Pain syndrome<br>Anemia	no	-	no	no change	yes	yes
Patient 7	2512	unknown	16.07.2019	M	64	OU	yes	2	NDM-1	10^3	Cornea injury	no	Conjunctivitis,<br>Keratitis<br>Uveitis	unknown	yes<br>(details assignment unknown)	unknown	yes	Left eye vision loss	yes	unknown
Patient 8	2024	29.07.18	01.08.18	M	nd	CU	no	1	no	10^5	B-cell lymphoma with the defeat of the retroperitoneal lymph nodes, liver, stomach, duodenum, pancreas, hepatic flexure of the colon, inferior vena cava and portal vein	yes	Febrile neutropenia.<br>Sepsis.<br>Stomatitis.	HD 2 st.<br>Cardiac risk 2 st.<br>Obstructive jaundice<br>CHC<br>Hepatic failure<br>LEVVD<br>Lower polyneuropathy<br>Radicular ischemia	1)Trimethoprim/sulfamethoxazole<br>2) Amoxycillin, Ciprofloxacin<br>3) Imipenem<br>4) Meropenem<br>5) Polymyxin B<br>6) Aztreonam, Ceftazidim\Avibactam	1) 29.07. - 27.08.18<br>2) 29-31.07.18<br>3)2-3.08.18<br>4)3-16.08.18<br>5) 08-16.08.18<br>6) 16-21.08.18	no	no change	unknown	yes

[a] Unit: CU - coloproctological unit; DPD - day patient department; AU - abdominal unit; RU - radiology unit; SBSTU - skin, bone and soft tissue tumors unit; OU - Ophthalmology unit; CU - chemotherapy unit;

[b] Hospital: 1 - Oncological Centre, St. Petersburg, Russia; 2 - City hospital №2, St. Petersburg, Russia;

[c] Comorbidity: CHD - coronary heart disease; AC - atherosclerotic cardiosclerosis; HD - hypertensive disease; DM - diabetes mellitus; MVD - mitral valve disease; CVD – cerebrovascular disease; AS - aortic stenosis; CHF - chronic heart failure; CHC - Chronic hepatitis C infection; LEVVD - lower extremity varicose vein disease
