# Supplementary material for: Emergence of Hybrid Resistance and Virulence Plasmids Harboring New Delhi Metallo-β-Lactamase in Klebsiella pneumoniae in Russia
Source: Antibiotics (Basel). 2021 Jun 9;10(6):691. doi: 10.3390/antibiotics10060691 (PMC8226487; doi:10.3390/antibiotics10060691)
Supplement: Supplementary file 1 [file antibiotics-10-00691-s001.zip › Supplementary Material Tab S2.pdf]

Table S2: Phenotypic and genotypic characteristics of twelve CR-hvKP (1657_kpn, 1659_kpn, 1970_kpn, 1971_kpn, 2024_kpn, 2471_kpn, 2501_kpn, 2512_kpn, 2566_kpn) obtained using hybrid genome assembly, in comparison with the previously reported isolates with hybrid plasmids KpvST147B_SE1_1_NDM, KpvST383_NDM_OXA-48, 51015.

**Isolate ID**	**1657_kpn**	**1659_kpn**	**1970_kpn**	**1971_kpn**	**2024_kpn**	**2471_kpn**	**2501_kpn**	**2512_kpn**	**2566_kpn**	**KpvST147B_SE1_1_NDM**	**KpvST383_NDM_OXA-48**	**51015**
**MLST**	395	147	147	395	395	874	15	395	147	147	383	15
**Accession numbers**	-	-	CP066855-CP066858	MW911666	MW911667	MW911668	MW911669	MW911670	MW911671	CP040724-CP040731	CP034200-CP034202	CP050376.1-CP050381.1
**Capsule type**	KL2	KL20	KL20	KL2	KL2	KL45	KL19	KL2	KL20	KL35	KL30	KL24
**O antigen**	O1v1	O2v1	O2v1	O1v1	O1v1	O1v2	O1/O2v2	O1v1	O2v1	O2v1	O1v2	O1v1
**Mouse lethality (LD_{50}, CFU)**	10^2	10^4	10^4	10^5	10^5	10^4	10^4	10^3	10^4	-	-	-
**Meropenem MIC**	64	32	8	16	4	4	8	4	8	-	-	-
**Chromosomal AMR genes**	oqxAB, fosA, $bla_{SHV-182}$	oqxAB, fosA, bla_{SHV-11}	fosA5, oqxAB, bla_{SHV-67}	oqxAB, fosA, $bla_{SHV-182}$	oqxAB, fosA, $bla_{SHV-182}$	oqxAB, fosA, $bla_{SHV-121}$	oqxAB, fosA, bla_{SHV-28}, aac(3)-IId	oqxAB, fosA, $bla_{SHV-182}$	oqxAB, fosA, bla_{SHV-11}	fosA5, oqxAB, bla_{SHV-67}	bla_{SHV-26}, catA1, fosA, tet(A), mph(A), oqxAB	fosA5, oqxAB, bla_{SHV}
**Chromosomal CRISPR**	-	type I-E CRISPR systems with 1 CRISPR array (43 spacers)	type I-E CRISPR systems with 1 CRISPR array (43 spacers)	-	-	-	type I-E* CRISPR systems with 1 CRISPR array (43 spacers)	-	type I-E CRISPR systems with 1 CRISPR array (43 spacers)	type I-E* CRISPR systems with 2 CRISPR array (10+9 spacers)	type I-E CRISPR systems with 1 CRISPR array (15 spacers)	type I-E* CRISPR systems with 2 CRISPR array (10+9 spacers)
**Chromosomal virulence genes**	entABES, fepBCDG, ecpABCDER, fimABCEH, ybtAEPQSTUX, fyuA, irp1, irp2, kdsA, silA	entABES, fepBCDG, ecpABCDER, fimABCEH, kdsA, silA	entABES, fepBCDG, ecpABCDER, fimABCEH, kdsA, silA	entABES, fepBCDG, ecpABCDER, fimABCEH, ybtAEPQSTUX, fyuA, irp1, irp2, kdsA	entABES, fepBCDG, ecpABCDER, fimABCEH, ybtAEPQSTUX, fyuA, irp1, irp2, kdsA	entABES, fepBCDG, ecpABCDER, fimABCEH, kdsA, silR	entABES, fepBCDG, ecpABCDER, fimABCEH, kdsA, silA	entABES, fepBCDG, ecpABCDER, fimABCEH, ybtAEPQSTUX, fyuA, irp1, irp2, kdsA	entABES, fepBCDG, ecpABCDER, fimABCEH, kdsA, silA	kdsA, ybtAEPQSTUX, irp1, irp2, fyuA, fimABCEH, ecpABCDER, fepBCDG, entABES	entABES, fepBCDG, ecpABCDER, fimABCEH, kdsA	ecpABCDER kdsA, ybtAEPQSTUX, irp1, irp2, fyuA, fimABCEH, ecpABCDER, fepBCDG, entABES, silA
**RM of non-mosaic plasmids**	-	-	-	-	-	FIB/FII: M.EcoJA03PI (type I)	FIB/FII: M.EcoJA03PI (type I)	-	-	FIB: S.Kpn1420II (type I), M.Kpn928I (type I)	-	-
**No. of plasmids**	1	3	3	2	4	2	2	1	2	7	2	5

**IncFIB/IncHI1B**	**AMR**	-	bla_{NDM-1}, mph(A), mph(E), msr(E), armA, aph(3')-VI, qnrS1, sul1, sul2, dfrA5	sul2, armA, msr(E), mph(E), qnrS1, aph(3')-VI, bla_{NDM-29}, dfrA5, sul1	bla_{NDM-1}, mph(A), mph(E), msr(E), armA, aph(3')-VI, qnrS1, sul1, sul2, dfrA5	-	bla_{NDM-1}, mph(A), mph(E), msr(E), aph(3')-VI, qnrS1, sul1, dfrA5	bla_{NDM-1}, mph(E), msr(E), armA, aph(3')-VI, qnrS1, sul2	bla_{NDM-1}, bla_{OXA-1}, $bla_{CTX-M-15}$, bla_{TEM-1B}, mph(A), mph(E), msr(E), armA, aph(3')-VI, aac(6')-Ib-cr, qnrS1, sul1, sul2, dfrA1, dfrA5, catA1, catB3, tet(A)	bla_{NDM-1}, mph(A), mph(E), msr(E), armA, aph(3')-VI, qnrS1, sul1, sul2, dfrA5	sul1, sul2, armA, dfrA5, mph(A), msr(E), mph(E), $bla_{CTX-M-15}$	bla_{NDM-5}, $bla_{CTXM-15}$, qnrS1, aac(6')-lb, bla_{OXA-9}, bla_{TEM-1B}, catA1, sul1, sul2, armA, dfrA5, aph(3')-1a, aph(3')-VI, aadA1, mph(A), mph(E), msr(E)	bla_{NDM-1}, qnrS1, dfrA5, sul1, sul2, armA, aph(3')-Ia, aph(3')-VI, mph(A), mph(E), msr(E)
	**virulence genes**	-	$_p$rmpA, $_p$rmpA2, iucABCD, iutA, terABCDEWXYZ, peg-344, pagO, shiF, ydjA, cobW, luxR	iutA, iucABCD, $_p$rmpA, $_p$rmpA2, peg-344, cobW, luxR pagO, ydjA, shiF	$_p$rmpA2, iucABCD, iutA, pagO, shiF, ydjA, cobW	-	$_p$rmpA, $_p$rmpA2, iucABCD, iutA, terABCDEWXYZ, peg-344, pagO, shiF, ydjA, cobW, luxR	$_p$rmpA, $_p$rmpA2, iucABCD, iutA, peg-344, pagO, shiF, ydjA, cobW, luxR	$_p$rmpA, $_p$rmpA2, iucABCD, iutA, terABCDEWXYZ, peg-344, pagO, shiF, ydjA, cobW, luxR	$_p$rmpA, $_p$rmpA2, iucABCD, iutA, terABCDEWXYZ, peg-344, pagO, shiF, ydjA, cobW, luxR	iutA, iucABCD, $_p$rmpA, $_p$rmpA2, peg-344, terABCDEWXYZ, cobW, luxR, pagO, ydjA, shiF	iutA, iucABCD, $_p$rmpA, $_p$rmpA2, peg-344, terABCDEWX, cobW, luxR, pagO, ydjA, shiF	iutA, iucABCD, $_p$rmpA, $_p$rmpA2, peg-344, terABCDEWXYZ, cobW, luxR, pagO, ydjA, shiF
**IncFIB/IncHI1B/IncR**	**AMR**	bla_{NDM-1}, bla_{TEM-1B}, mph(A), aph(3')-VI, qnrS1, sul1, dfrA1, dfrA5, catA1, tet(A)	-	-	-	qnrS1, aac(6')-Ib-cr, tet(A), dfrA1, sul1, catA1, catB3, $bla_{CTX-M-15}$, bla_{OXA-1}, bla_{TEM-1B}	-	-	-	-	-	-	-
	**virulence genes**	$_p$rmpA, $_p$rmpA2, iucABCD, iutA, terABCDEWXYZ, peg-344, pagO, shiF, ydjA, cobW	-	-	-	iucA, $_p$rmpA, peg-344, luxR, shiF	-	-	-	-	-	-	-
**AMR genes of IncFIB**		-	-	dfrA5	-	-	-	-	-	-	aac(6')-Ib-cr, bla_{OXA-1}. catB3, arr-3, sul1, $bla_{CTX-M-15}$, qnrS1, aph(3')-VI, bla_{NDM-1}	-	-
**AMR genes of IncR**		-	bla_{OXA-9}, $bla_{CTX-M-15}$, bla_{TEM-1A}, aac(6')-Ib, aadA1, qnrS1; **virulence factors:** merACDEPRT	bla_{TEM-1A}, bla_{OXA-9}, aadA, aac(6')-Ib	bla_{OXA-1}, $bla_{CTX-M-15}$, bla_{TEM-1B}, aac(6')-Ib-cr, qnrS1, sul1, dfrA1, catA1, catB3, tet(A)	-	-	-	-	$bla_{CTX-M-15}$, qnrS1; **virulence factors:** merACDEPRT	aac(6')-Ib, aadA1, bla_{OXA-9}, bla_{TEM-1A}	-	-
**AMR genes of IncL**		-	-	-	-	-	-	-	-	-	-	aph(6)-Id, aph(3')-Ib,	-

												aph(3’)-VIb, $bla_{\text{CTX-M-14b}}$, $bla_{\text{OXA-48}}$	
**AMR of IncFIB/IncFII**	**AMR**	-	-	-	-	-	$bla_{\text{OXA-1}}$, $bla_{\text{CTX-M-15}}$, aph(3')-Ia, aac(6')-Ib-cr, catA1, catB3	$bla_{\text{OXA-1}}$, $bla_{\text{CTX-M-15}}$, $bla_{\text{TEM-1B}}$, mph(A), aac(6')-Ib-cr, aadA2, sul1, dfrA12, catB3, tet(A)	-	-	-	-	aac(3)-IId, mph(A), sul2, aph(3')-Ib, aph(6)-Id, catB3, $bla_{\text{OXA-1}}$, aac(6')-Ib-cr, $bla_{\text{CTX-M-15}}$, aph(3')-Ia, $bla_{\text{TEM-1B}}$
	**virulence genes**					-	arsABCDR, pcoABCDERS, silABCEFGPRS	arsABCDR, pcoABCDERS, silABCEFGPRS					arsABCDR, pcoABCDERS, silABCEFGPRS,
**Other plasmids**		-	ColRNAI	-	-	ColRNAI	-		-	-	IncFIB, IncR, IncX4	-	IncFII, ColRNAI
