# Supplementary material for: Emergence of Hybrid Resistance and Virulence Plasmids Harboring New Delhi Metallo-β-Lactamase in Klebsiella pneumoniae in Russia
Source: Antibiotics (Basel). 2021 Jun 9;10(6):691. doi: 10.3390/antibiotics10060691 (PMC8226487; doi:10.3390/antibiotics10060691)
Supplement: Supplementary file 1 [file antibiotics-10-00691-s001.zip › Supplementary Material Tab S4.pdf]

Table S4: MICs for the NDM variants under standard and zinc-limiting conditions (50 µM EDTA). Shown are the MICs (mg/L) for β-lactams of the clinical isolate 1970_kpn of Kpn, *E. coli* XL10-Gold, *E. coli* XL10-Gold harboring *bla*$_{NDM-1}$ and *bla*$_{NDM-29}$.

Condition	Standard conditions							50 µM EDTA					
Variant	1970_ kpn	pJET1.2/ NDM-1	pJET1.2/ NDM-29	pEGFP-N3/ NDM-1	pEGFP-N3/ NDM-29	pEGFP-N3	XL10-Gold	1970_ kpn	pJET1.2/ NDM-1	pJET1.2 / NDM-29	pEGFP -N3/ NDM-1	pEGFP -N3/ NDM-29	pEGFP -N3
Ampicillin	>512	>512	>512	>512	>512	0,25	2	>512	>512	>512	4	4	0,25
Cefotaxime	128	256	128	256	256	<0,125	<0,13	<0,13	0,25	0,25	<0,13	<0,13	<0,125
Cefoxitin	>64	>64	>64	>64	>64	-	0,5	2	8	8	8	4	-
Ceftriaxone	256	256	256	256	256	<0,125	<0,13	<0,13	0,25	0,25	<0,13	<0,13	<0,125
Ceftazidime	>256	>256	>256	>256	>256	0,5	1	4	2	4	4	8	0,5
Cefepime	16	64	32	32	32	<0,125	<0,13	0,25	0,25	0,13	0,13	0,13	<0,125
Aztreonam	0,13	0,25	0,13	0,13	<0,06	0,5	0,13	<0,02	0,03	0,03	<0,02	<0,02	0,5
Meropenem	8	8	8	8	8	<0,125	<0,02	0,25	0,13	0,13	0,06	0,13	<0,125
Ertapenem	>32	>32	>32	>32	>32	<0,25	0,06	<0,13	<0,13	<0,13	<0,13	<0,13	<0,25
Biapenem	4	0,5	1	1	0,25	0,03	0,06	0,5	0,06	0,06	0,03	0,03	0,03
Gentamicin	128	<2	<2	64	32	>32	<2	-	-	-	-	-	>32
Amikacin	>256	1	1	1	0,5	1	0,25	-	-	-	-	-	1
Ciprofloxacin	128	<1	<1	<1	<1	0,25	<1	-	-	-	-	-	0,25
Piperacilline/ Tazobactam	>512	512	512	256	512	-	0,5	>512	256	256	0,5	1	-
Polymyxin B	<0,03	<0,03	<0,03	<0,03	<0,03	<0,25	<0,03	<0,03	<0,03	<0,03	<0,03	<0,03	<0,25
