# Supplementary material for: Emergence of Hybrid Resistance and Virulence Plasmids Harboring New Delhi Metallo-β-Lactamase in Klebsiella pneumoniae in Russia
Source: Antibiotics (Basel). 2021 Jun 9;10(6):691. doi: 10.3390/antibiotics10060691 (PMC8226487; doi:10.3390/antibiotics10060691)
Supplement: Supplementary file 1 [file antibiotics-10-00691-s001.zip › Supplementary Material Text S1.pdf]

Text S1: Cloning of *bla*$_{NDM-29}$ and *bla*$_{NDM-1}$

The 1139 bp DNA fragment carrying NDM-29 MBL together with its native promoter was amplified using primers NDM-dw (5'-CTGGGTCGAGGTCAGGATAG-3') (Liu et al. 2018) and R_NDM_clon-reverse (5'-TTTGCTCAGCTTGTTGATTATC-3') (this study). Then the fragment was cloned at NdeI and XbaI restriction sites in pJET1.2 vector (CloneJET PCR Cloning Kit, Life Technologies), that contains β-lactamase gene (ampicillin resistance), resulting in recombinant plasmid pJET1.2/NDM-29. The recipient cells of XL10-Gold *Escherichia coli* strain {endA1 glnV44 recA1 thi-1 gyrA96 relA1 lac Hte Δ(mcrA)183 Δ(mcrCB-hsdSMR-mrr)173 tetR F'[proAB lacIqZΔM15 Tn10(TetR Amy CmR)]} (Stratagene, United Kingdom) were chemically transformed as previously described (Maniatis Tom, Sambrook Joseph 1982). The same manipulations were performed with *bla*$_{NDM-1}$ gene that does not contain any AAS. *bla*$_{NDM-29}$ and *bla*$_{NDM-1}$ were also cloned into the pEGFP-N3 expression vector (Clontech Co., Palo Alto, CA, USA), conferring resistance to kanamycin, at XhoI and HindIII restriction sites. Another type of transformant was XL10-Gold *Escherichia coli* with pEGFP-N3 expression vector without the insert. Sanger sequencing was used to confirm the cloned inserts of the pEGFP-N3 cloning vector using primers NDM-F-forward (5'-GGTTTGGCGATCTGGTTTTC-3') (Nordmann, Naas, and Poirel 2011), M-NDM-R-reverse (5'-TGCTCAGTGTCGGCATCACC-3') (this study), and pJET1.2 forward sequencing primer (5'- CGACTCACTATAGGGAGAGCGGC-3'), pJET1.2 reverse sequencing primer (5'- AAGAACATCGATTTTCCATGGCAG-3') (CloneJET PCR Cloning Kit, Life Technologies) to confirm the cloned inserts of the pJET1.2 cloning vector.
